# Less is more: Depleting myeloid‐biased HSCs to restore immune function

**DOI:** 10.1002/hem3.125

**Published:** 2024-07-19

**Authors:** Hansen J. Kosasih, Charles E. de Bock

**Affiliations:** ^1^ Children's Cancer Institute, Lowy Cancer Research Centre UNSW Sydney Kensington New South Wales Australia; ^2^ School of Clinical Medicine UNSW Sydney Kensington New South Wales Australia

The quest to unlock the secrets of eternal youth or extending life span have been described throughout history. This includes ancient texts describing the “fountain of youth” and more recently within the popular fiction series “Harry Potter” where the philosopher's stone provides an “elixir of life.” In reality, our longevity is in part due to the presence of an effective immune system coupled with our ability to pre‐emptively manipulate this using vaccinations. Indeed, it could be argued that vaccinations remain one of the most successful health interventions in human history, consigning many of the debilitating illness such as smallpox to the annals of history. Nevertheless, there remains an ongoing need for the rapid development and deployment of new vaccines to protect ourselves against new and emerging threats such as COVID‐19. However, whilst we can design new vaccines based on an exquisite understanding of the “enemy,” this needs to be coupled with an individual's ability to mount an effective immune response—which, unfortunately, declines as we age.^1^

It is now established that the hematopoietic stem cell (HSC) population changes over time.^2^ In youth, the HSCs population has a balanced output of lymphoid and myeloid cells (bal‐HSCs), but then changes towards myeloid‐biased HSCs (my‐HSCs) in older individuals. This in turn results in decreased lymphopoiesis, increased myelopoiesis as well as proinflammation, myeloid‐related malignancies and a reduced adaptive immune response in older individuals.^3^ An elegant new study published in Nature by the Weissman lab^4^ now provides a tantalising new approach to improve our immune response. They demonstrate that an antibody depletion‐based strategy targeting my‐HSCs, can push the immune system in favor of a more balanced HSCs, and in essence, reversing time to rejuvenate an old immune system to a more youthful age.

Previous studies which have characterised the HSC population (Lin^−^, SCA1^+^ KIT^+^ FLT3^−^ CD34^−^), found that my‐HSCs have higher expression of CD150 (*Slamf1*) compared to bal‐HSCs.^3^ In this new study, Ross et al.^4^ sought to extend this by using transcriptional datasets to find the best set of cell surface markers that identify my‐HSCs for antibody targeting. They found that the most highly enriched cell‐surface markers in my‐HSCs were CD41 (*Itga2b*), CD62p (*Selp*), and NEO1 (*Neo1*). These were then validated using flow cytometry on CD150^high^ HSCs (my‐HSCs) vs. CD150^low^ HSCs (bal‐HSCs) and found that in older mice, the proportion of HSCs that were NEO1+, CD41+, and CD62p+ increased, consistent with the observed increase in my‐HSCs associated with aging. These markers were also largely limited to HSCs except for CD41 that was also found on megakaryocyte progenitors. Therefore, the authors settled on NEO1, CD62p, together with CD150, as candidate targets for my‐HSC depletion in vivo.

To remove my‐HSCs in vivo, the authors tested different cocktails combining antibodies that target CD150, CD62p, NEO1 as well as KIT and CD47 that blocks any antiphagocytic signal (Figure 1). Using either anti‐CD62p or anti‐NEO1 or anti‐CD150 together with anti‐KIT and anti‐CD47 antibodies, they were able to effectively decrease my‐HSCs in the bone marrow 1 week after injection. Functionally, the remaining HSCs were found to have gene signatures of young HSCs and result in a low myeloid‐to‐lymphoid cell ratio when transplanted into secondary recipients Further, mice that underwent antibody conditioning were found to have significantly higher levels of circulating T and B cells and lower levels of proinflammatory mediators compared to age‐matched controls. The question was then whether this also equated with enhanced immune function? To test this, my‐HSC‐depleted mice were challenged with live‐attenuated mouse Friend retrovirus. Excitingly, this resulted in increased virus‐specific CD8^+^ T cells and mice maintained a robust immune response when re‐challenged 6 weeks after the initial vaccination.

**Figure 1:**
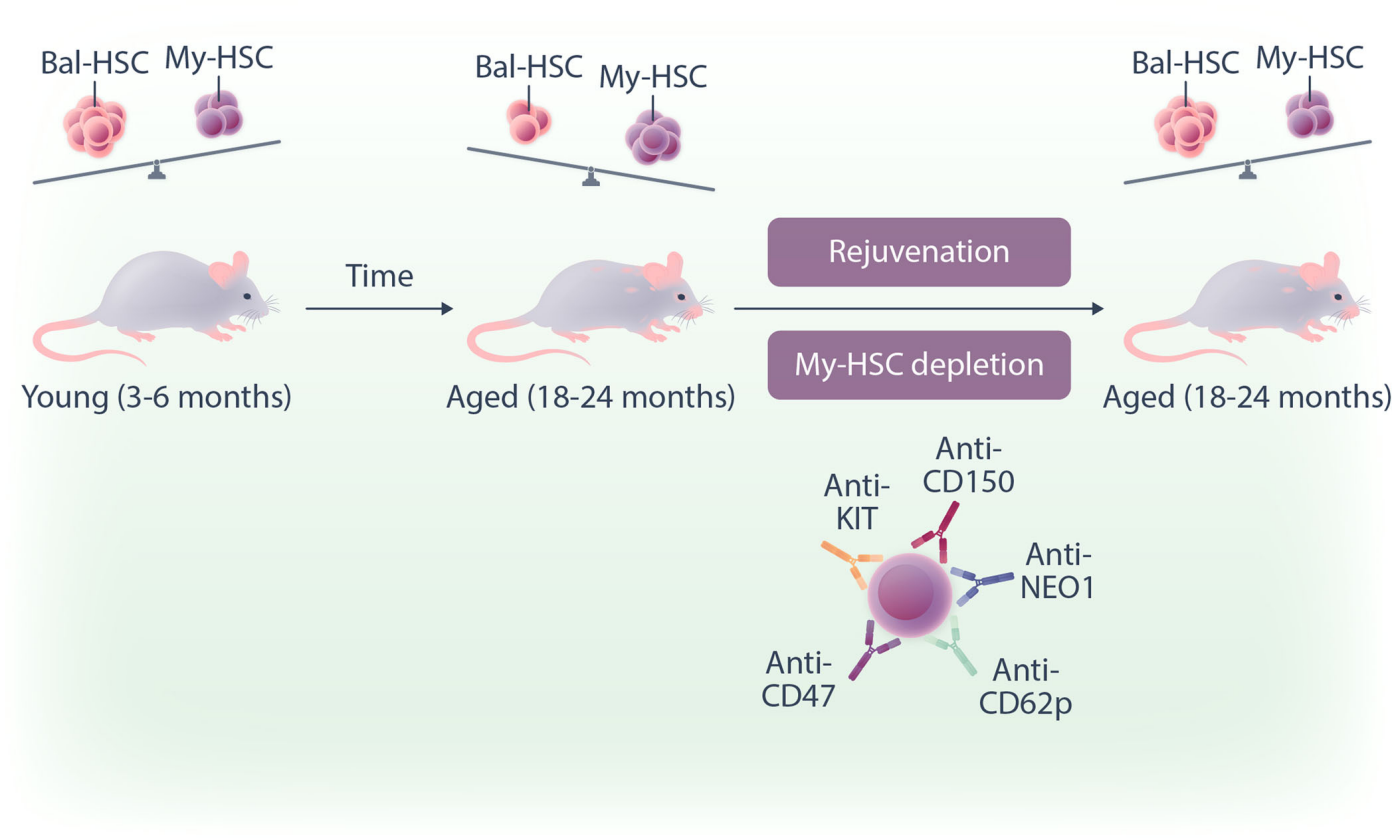
Antibody mediated depletion of myeloid biased hematopoietic stem cells (HSCs) to restore immune function. As mice age, their HSC pool changes to favor myeloid output from myeloid‐biased HSCs (my‐HSCs) over balanced HSCs (bal‐HSCs) that contribute to both lymphoid and myeloid lineages. Treating mice with an antibody cocktail targeting CD150 NEO1 or CD62p along with both KIT and CD47 selectively depletes the my‐HSC pool and restores the immune system to that found in younger mice.

Can these findings now be translated to the clinic in the future? The authors provide preliminary data that several genes for mouse my‐HSCs are also enriched in humans HSCs associated with age including *SELP* (CD62p), *SLAMF1* (CD150), and *NEO1* (NEO1) suggesting the same my‐HSC depletion strategy should also theoretically restore immune function in humans. Another attractive prospect for my‐HSC depletion might be in the context of allogeneic HSC transplantation (allo‐HSCT). In conventional allo‐HSCT, the donor receives high doses of chemotherapy and/or irradiation to improve the stable engraftment of donor HSCs. Unfortunately, this is associated with a range of side effects. To avoid the need for irradiation/chemotherapy, new pre‐clinical studies have used antibody‐based preconditioning methods to target hematopoietic stem and immune cells with minimal overall toxicity.^5^ Therefore, antibody preconditioning could be used on both donors and recipients. For donors, antibody preconditioning is used to remove my‐HSCs prior to donation to not only improve the quality of the donor HSC pool but also allow an older population to act as prospective donors. Taken together, the prospect of rejuvenating the immune system coupled with medical advances, such as the development of new vaccines, might be the key to uncovering philosopher's stone.

## AUTHOR CONTRIBUTIONS

Hansen J. Kosasih and Charles E. de Bock conceptualized and co‐wrote the article. Both authors agreed to the final version.

## CONFLICT OF INTEREST STATEMENT

The authors declare no conflict of interest.

## FUNDING

No funding was received for this publication.

## Data Availability

Data sharing not applicable to this article as no data sets were generated or analyzed during the current study.
